# A prospective pilotstudy comparing the anesthetic effects of an alpha-2 agonist during holmium laser resection of the prostate and transurethral resection for prostate surgery for benign prostatic hyperplasia patients using selective alpha-1 blockers

**DOI:** 10.1186/s12871-018-0598-1

**Published:** 2018-09-27

**Authors:** Do-Won Lee, Jiseok Baik, Giyoung Yun, Soeun Jeon, Hyae-Jin Kim, Eun-Soo Kim, Hyeon Jeong Lee, Jae-Young Kwon

**Affiliations:** Department of Anesthesia and Pain Medicine, School of Medicine, Pusan National University, Biomedical Research Institute, Pusan National University Hospital, 179 Gudeok-ro, Seo-gu, Busan-si, 49241 South Korea

**Keywords:** Dexmedetomidine, α2 agonist, α1 antagonist, Holmium laser resection of the prostate (HoLEP), Transurethral resection of prostate (TURP), Benign prostatic hyperplasia (BPH)

## Abstract

**Background:**

To examine the response to an α2receptor agonist used as a sedative for patients using long-term selective α1 blockers.

**Methods:**

Sixty-nine consecutive patients undergoing transurethral prostate resection or holmium laser resection of the prostateunder spinal anesthesia were divided into two groups; group N (*n* = 37), which did not receive α1 blockers, and group T (*n* = 32), which was administered tamsulosin for at least 1 month before the study. Bispectral index scores, Modified Observer’s Assessment of Alertness/Sedation scale scores, heart rate, and mean blood pressure were obtained under sedation using dexmedetomidine for 30 min during surgery.

**Results:**

The only significant difference found between the groups were mean bloodpressure 15 min after the first loading dose injection of dexmedetomidine. Differencesbetween both groupswere noted at 15 min(group T: 100.2 ± 12.9 mmHg; group N: 90.0 ± 17.5 mmHg; *P* = 0.08), 20 min (group T: 99.8 ± 12.3 mmHg; group N: 87.4 ± 15.0 mmHg; *P* < 0.00), 25 min (group T: 99.3 ± 13.4 mmHg; group N: 85.4 ± 13.8 mmHg; P < 0.00), and 30 min (group T: 98.8 ± 13.1 mmHg; group N: 84.5 ± 13.5 mmHg; P < 0.00).

**Conclusions:**

The use of α2 agonists is appropriate during surgery for benign prostatic hyperplasia patients using tamsulosin, and there is no need to alter the dose. Alertness with anesthesia involving α2 agents was maintained for patients using long-term tamsulosin and patients who did not use tamsulosin.

**Trial registration:**

The study was retrospectively registered with the Clinical Research Informational Service (KCT0002967, July 2, 2018).

**Electronic supplementary material:**

The online version of this article (10.1186/s12871-018-0598-1) contains supplementary material, which is available to authorized users.

## Background

Selective α1 blockers are commonly used for the medical treatment of lower urinary tract obstruction in patients with benign prostatic hyperplasia(BPH); however, they may cause side effects such as asthenia, fatigue, postural hypotension, and dizziness [1]. Long-acting α1 blockers commonly used for these patients with BPH include terazosin, doxazosin, silodosin, tamsulosin, and naftofidil. The effectiveness of these drugs compared with placebo has been proven in several studies [2–6]. These α blockers are selective α1 blockers and are different compared to nonspecificα blockers such as phenoxybenzamine, which has been used previously. Phenoxybenzamine has a high affinity for α2 adrenoceptors, which are found in the prostatic neuroeffector cleft [7, 8].Tamsulosin is the first clinically available α1Aadrenoceptor blocker [9]. It is selective for α1A adrenoceptors, which are predominantly present and functional in the human prostate, and it is more selective for α1 adrenoceptors in the human prostate than in the human aorta [1].In patients with BPH symptoms, tamsulosin has been shown to relieve symptoms and improve blood flow, andunlike terazosin and doxazosin, blood pressure is not significantly affected [9].

Despite medical treatment with these elective α1 blockers, many patients have indications for surgical resection such as transurethral resection of the prostate (TURP) or Holmium laser resection of the prostate (HoLEP). These surgical resections are relatively simple and do not cause major complications. Therefore, they can be effective for quick surgeries. However, along with surgical risks, there is always an anesthetic risk. Because BPH is a common condition affecting men older than 45 years [10], 80% will develop BPH symptoms by age 80 years, and it is estimated that 90% of men 80 to 89 years of age exhibit histologic evidence of disease [9, 11, 12]. In other words, many patients who need surgery are older, and anesthesia used for these elderly patients requires more attention and preparation. In general, elderly patients can expect a high incidence of intraoperative and postoperative complications and increased mortality, even if they do not have underlying disease [13]. In general, morbidity rates (51% vs 28%) and mortality rates (7% vs 2.3%) are higher for those 80 years or older [13]. Hong et al. [14] reported that the use of dexmedetomidine (DMT) required longer postanesthetic care unit (PACU) stays compared to those in the control group comprising of patients who were 65 years and older which used normal saline and were administered spinal anesthesia with low dose of bupivacaine.

For these elder patients undergoing surgical resection such as TURP and HoLEP, DMT sedation with spinal anesthesia has several beneficial actions during the perioperative period. DMT, which is an α2 adrenoreceptor agonist, decreases sympathetic tone and attenuates the neuroendocrine and hemodynamic responses to anesthesia and surgery, reduces anesthetic and opioid requirements, and causes sedation and analgesia [15]. DMT was approved by the Food and Drug Administration (FDA) in 1999 as an analgesic agent and sedative for use in the intensive care unit (ICU) [15]. In particular, DMT has been used for ICU patients when daily assessment is done for ventilator weaning, extubation or neurological examination.Since its approval, DMT has been widely used not only in the ICU but also in the operating room owing to its analgesic and sedative effects. DMT is a selective α2 adrenergic agonist that affects the locus ceruleus area, which is related to the modulation of sleep regulation and respiratory control. The α1 adrenoceptorswhich are involved in motor activity have been found to act in a number of brain regions, including the locus ceruleus, dorsal raphe, vermis cerebellum, medial preoptic area, nucleus accumbens, motor cortex, and piriform cortex, and it is probably involved in the first cervical nucleus of the ventrolateral medulla, the ventral tegmental area, lateral hypothalamus, and prefrontal cortex [16]. Interestingly, there are substantial dense concentrations of α2 and α1 adrenoceptors in the locus ceruleus area [17, 18]. Virtanen et al. [19] reported that the central α1 and α2 adrenoceptor activities were observed at higher dose of DMT (> 1000 μg / kg) or in rapid infusion of lower dose of DMT.Central α1 and α2 adrenoceptors of different brain regions are known to have opposing behavioral activity, with one stimulating activity and the other inhibiting activity [20].

No study has elucidated the differences in the use of a transient α2 adrenoceptor agonist for sedation of patients using a nonspecific α blocker and a selective α1 blocker over a prolonged period of time compared to those who did not. This was the primary focus of this studyand in addition, the response to the α2 adrenoreceptor agonist used as a sedative by patients who were using long-term selective α1 blockers was examined.

## Methods

This study was conducted at our hospital and was approved by theInstitutional Review Board of our hospital (approval no: H-1804-014-066) and was registered with the Clinical Research Informational Service (KCT0002967, http://cris.nih.go.kr). Written informed consent was obtained from all patients. Eighty patients 45–80 years of age with an American Society of Anesthesiologists physical status of I or II who were scheduled to undergo HoLEP and TURP under spinal anesthesia and sedation using DMT were considered for enrollment in one of two groups: group N (No medication) and group T (Tamsulosin). Patients who were recommended to undergo surgery becauseof severe grade International Prostate Symptom Score (IPSS) or the large volume of prostate and high IPSS patients even with 0.4 mg of tamsulosin for 1 month or more were included in this clinical study. Exclusion criteria included body mass index more than 33 kg/m^2^. Patients with a psychiatric illness, any medical or surgical history involving the brain, any neurologic disorder, or those whoused antipsychotic drugs or sedatives were excluded from the study. Demographic data were recorded.

Baseline hemodynamic variables were noted using noninvasive measurement of mean blood pressure (MBP), heart rate (HR), oxygen saturation, end-tidal carbon dioxide, electrocardiogram results, and temperature.Before spinal anesthesia, crystalloid 10 mL/kg was administered intravenously for hydration. The patient was placed in the lateral recumbent position and a spinal tap was performed at L3/4 level. Hyperbaric bupivacaine 10 mg was administered intrathecally. The patient was then placed in the supine position, and oxygen 3 L/min was administered via a nasal prong. After confirmation of successful spinal anesthesia, a loading dose of DMTof 1.0 μg/kg was administered intravenously for 10 min followed by an infusion of DMT 1.0 μg/kg/h to patients in all groups.The syringe pump was operated by an anesthetist who is blinded in this study and the bispectral index (BIS) (A-2000 BIS monitor; Aspect Medical Systems Inc., Natick, MA, USA) [21] scores (Table 1(a)) and Modified Observer’s Assessment of Alertness/Sedation (MOAA/S) scale scores (Table 1(b)) were recorded 5 min after DMT administration [22]. Because the MOAA/S scale measurements may affect the BIS score, the BIS score was measured first. Patients were considered conscious if their MOAA/S scale scores were 5 (alert), 4, or 3; they were considered unconscious if the scores were 2, 1, or 0 (no response) (Table 1(b)). The depth of sedation was evaluated using the BIS. Table 1(a) shows a scale that associates the BIS score with the sedation degree [23]. BIS scores were recorded by an aspect monitor with surface electrodes. After wiping the skin with alcohol and drying, sensors were placed diagonally on the forehead as follows: one at the center of the forehead approximately 2 in. above the bridge of nose; one directly above the forehead; and one on the temple between the corner of the eye and hairline.

**Table 1:** BIS range (a), Responsiveness scores of the Modified Observer’s Assessment of Alertness/Sedation (MOAA/S) scale (b)

(a)	
BIS	Sedation degree
90–100	Awaken
70–89	Light to moderate sedation
60–69	Superficial anesthesia
45–59	Adequate anesthesia
0–45	Deep anesthesia
(b)	
Score	Response
5	Responds readily to name spoken in normal tone
4	Lethargic response to name spoken in normal tone
3	Responds only after name is called loudly or repeatedly
2	Responds only after mild prodding or shaking
1	Does not respond to mild prodding or shaking
0	Does not respond to noxious stimulus

This chart reflects a general association between clinical state and BIS values. These goals and their associated BIS scores may vary overtime and in the context of patient status and treatment plan

The first time the BIS score ranged between 80 and 89 was called BIS_80_, and the time from the loading of the drug was measured and recorded in seconds. BIS_70_ and BIS_60_ were measured in the same manner. The times to reach BIS_60_, BIS_70_, and BIS_80_ for each group were compared with each other. BIS_60_, BIS_70_, and BIS_80_ were compared between group N and group T. BIS scores and MOAA/S scale scores were measured at 5-min intervals (0, 5, 10, 15, 20, 25, and 30 min) from the beginning of the loading dose for all groups.

The BIS, MBP, HR and MOAA/S were manually recorded by a researcher. The HR and MBP of all patients were measured. The frequency of drug use and incidence of bradycardia and hypotension were recorded. If bradycardia ensued (HR less than 50), atropine 0.5 mg was administered intravenously. IfMBP was less than 50 mmHg, ephedrine 5 mg and crystalloid 300 mL were administered intravenously. Patients who had more than three bradycardia or hypotension events were excluded from the experimental group and aggressive medication was administered.

This study was designed as a prospective pilot study. The primary outcome variable was the first time (sec) of BIS_80_ after DMT administration. The mean value of BIS_80_ was considered to be significant when the difference was 10%. We also hypothesized that the long-term use of alpha 1 blockers would have little or no effect on the sedation rate between the two groups. Therefore, if the effective size is small or medium, the standardized effective size was set 0.5 [24]. Thus, the calculated number of samples required 32 patients per group. Then the result has a 95% confidence limit. Thus, 40 subjects were finally included per group to accommodate an expected loss of 20%.

Statistical analysis was performed using SPSS version 18 (SPSS Inc., Chicago, IL, USA). Quantitative variables were compared using an unpaired t-test between two groups. Student’s t-test was used to compare results for parametric data at each time point. All data are expressed as means (standard deviation) or numbers. *P* < 0.05 was considered statistically significant.

## Results

**Fig. 1:**
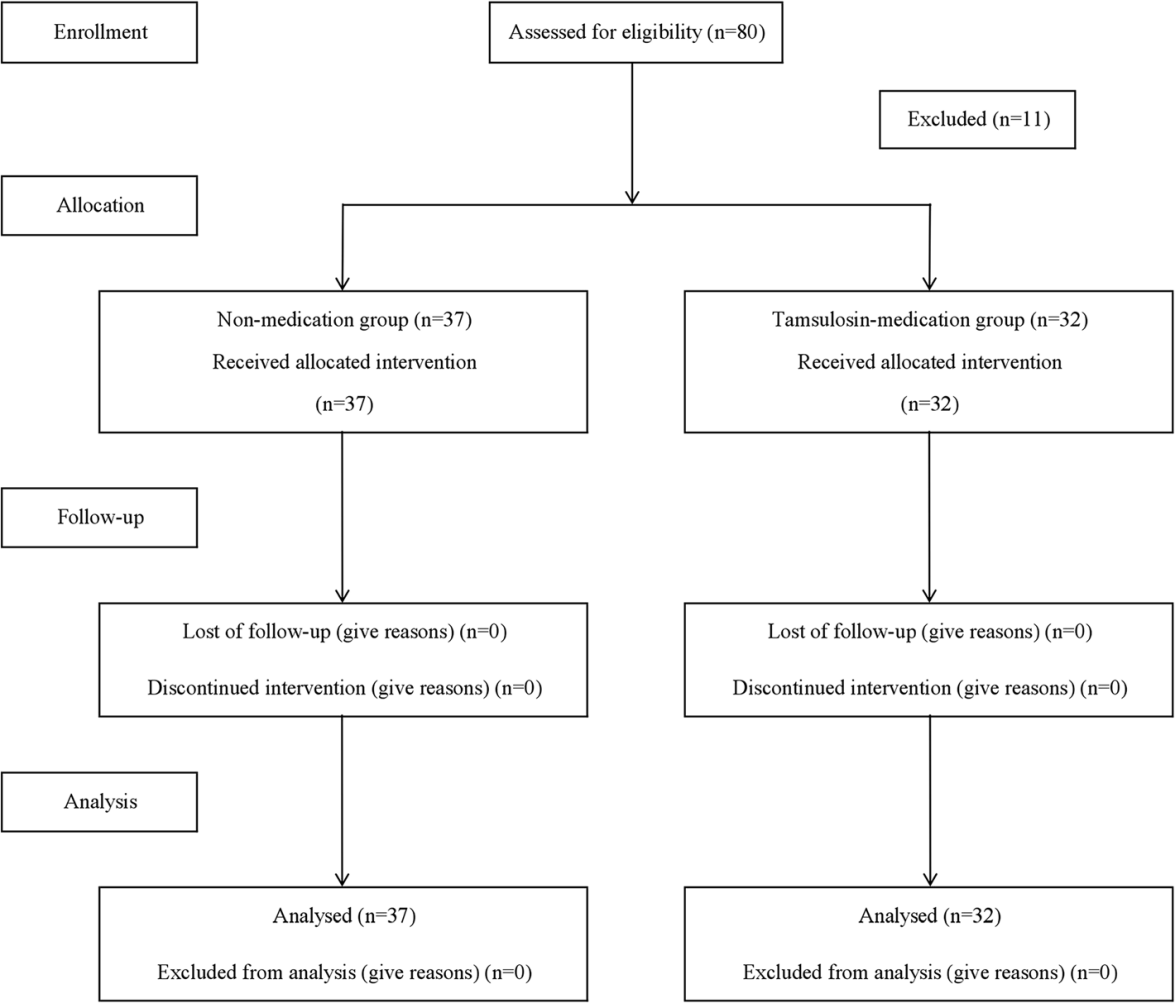
CONSORT flow diagram

A total of 80 patients were enrolled into two groups. Eight patients in Group T and three patients in Group N were excluded because of inappropriate spinal anesthesia. Thus, 69 patients completed the study (group *T* = 32; group *N* = 37) (Fig. 1). Demographic and perioperative data and duration of tamsulosin use are shown in Table 2. No significant differences were observed between the two groups regarding age, sex, height, weight, American Society of Anesthesiologists (ASA) classification, or type of surgery.

**Table 2:** Demographic data and patient characteristics

	Group N (*n* = 37)	Group T (*n* = 32)	*p*-value
Age (yr)	63.3 ± 8.2	65.5 ± 7.3	0.253
Height (cm)	168.4 ± 7.4	167.5 ± 4.6	0.541
Weight (kg)	69.5 ± 10.3	68.5 ± 6.8	0.625
Duration of tamsulosin use (month)	0	30.3 ± 28.5	ns
Type of Operation			
TURP	15	6	ns
HoLEP	22	26	ns
ASA class			
I	7	6	ns
II	30	26	ns

Results are expressed as means (SD) or numbers of patients. There are no significant statistical differences

**Fig. 2:**
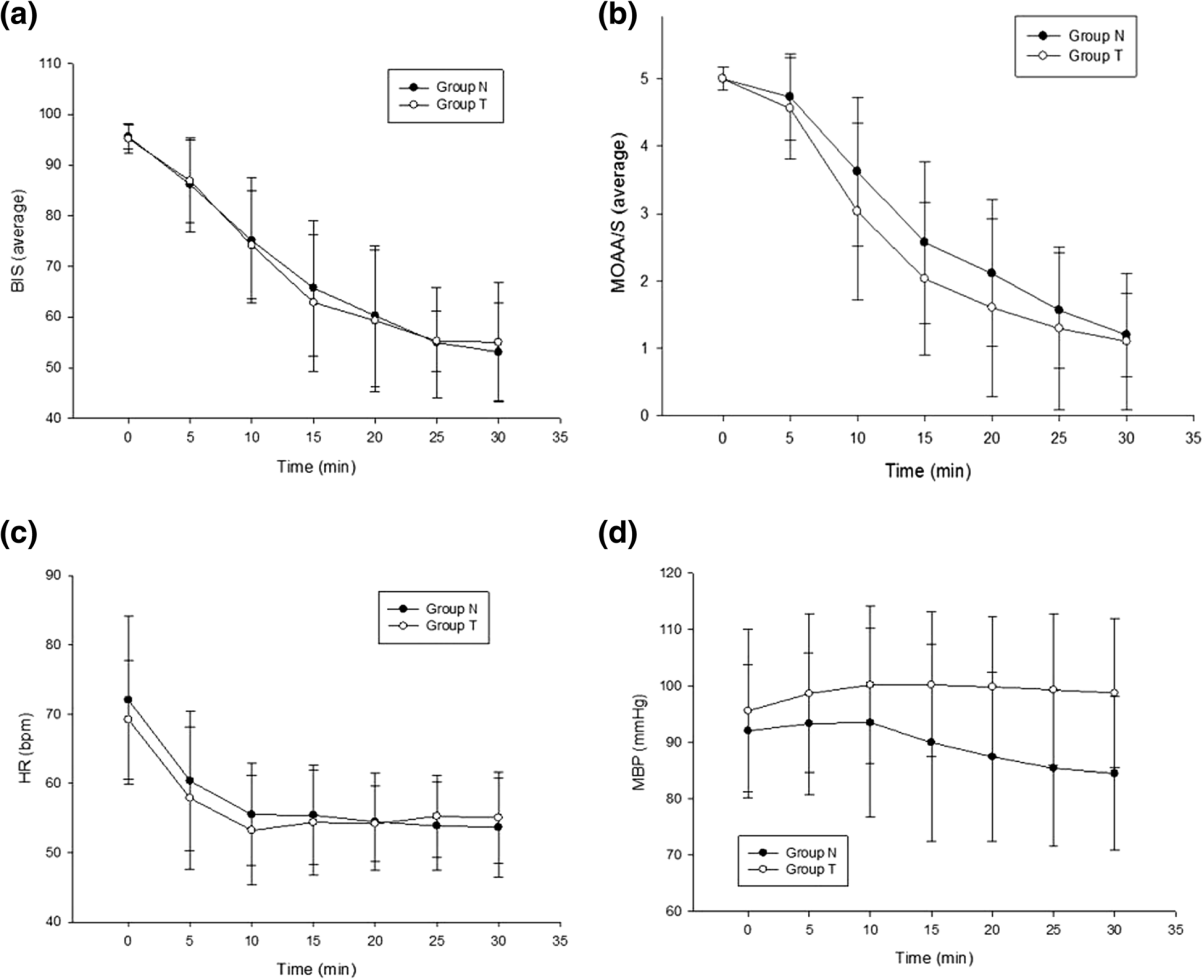
Intraoperative changes (5-min interval) in the bispectral index (BIS) (**a**), Modified Observer’s Assessment of Alertness/Sedation (MOAA/S) scale (**b**), heart rate (HR) (**c**), and mean blood pressure (MBP) (**b**) after beginning the loading infusion of dexmedetomidine (DMT). Data are presented as mean ± standard deviation

There was no statistically significant difference between the two groups regarding the BIS score measured from the beginning of use to 30 min later (Fig. 2(a)). The time periods required toachieveBIS_80_, BIS_70_, and BIS_60_were also not statistically significant (Table 3).

**Table 3:** BIS_80_, BIS_70_, and BIS_60_

Variables	Group N (*n* = 37)	Group T (*n* = 32)	*p*-value
BIS_80_	293.4 ± 1703	2333 ± 156.0	0.133
BIS_70_	483.4 ± 270.6	503.7 ± 294.7	0.767
BIS_60_	720.4 ± 3533	655.4 ± 290.7	0.411

Data are presented as mean ± standard deviation. The first time the BIS score ranged between 80 and 89 after the initial loading infusion was called BISso. The first time the BIS score ranged between 70 and 79 after the initial loading infusion was called BIS_70_. The first time the BIS score ranged between 60 and 69 after the initial loading infusion was called BIS_60_

There was no statistically significant difference between the two groups regarding MOAA/S scale scores measured from the beginning of use to 30 min later (Fig. 2(b)). In addition, no statistically significant difference between the HR results and the seven measurements of the results from the beginning of use to 30 min later (Fig. 2(c)). However, MBP of the group T was significantly higher than it of the group N at 15 min (group T: 100.2 ± 12.9 mmHg and group N: 90.0 ± 17.5 mmHg; *P* = 0.08), 20 min (group T: 99.8 ± 12.3 mmHg; group N: 87.4 ± 15.0 mmHg; *P* < 0.00), 25 min (group T: 99.3 ± 13.4 mmHg; group N: 85.4 ± 13.8 mmHg; *P* < 0.00), and 30 min (group T: 98.8 ± 13.1 mmHg; group N: 84.5 ± 13.5 mmHg; *P* < 0.00) (Fig. 2(d)).

## Discussion

The use of α1 blockers is an established therapy for patients with BPH. In addition, α1 blockers with increased selectivityhave been developed due to the efforts of many researchers to reduce side effects. The use of DMT in the operating room has been established not only as a supplementary agent for patients under general anesthesia but also as a light sedative drug for patients under regional anesthesia such as spinal anesthesia and epidural anesthesia. However, many cliniciansare unawareofthe interaction between these two drugs or their effects on one another. Our results confirmed that use of anα1 inhibitor for 1 month does not significantly affect the use of α2 agonists. However, this does not mean that the use of α1 inhibitors does not alter the α2 receptors in the brain. We believe that long-term use of α1 inhibitors is likely to affect α2 receptors, as already demonstrated by other studies [17–20]. Their capacity may not be as high because they affect brain receptors, or they may have had few effects on brain receptors due to the specificity of tamsulosin. Due to this uncertainty, we believe it is necessary to clarify this issue using animal experiments. However, our results were measured by administering a dose of α2 agonists used in the clinical setting. In other words, no problems inducing appropriate sleep or light anesthesia with α2 agonists have been found in patients who have been using α1 blockers in clinical practice. Based on these results, it is appropriate to use α2 agonists for surgery for BPH patients, and there is no need to increase or decrease the dose.

An appropriate BIS score to avoid recall in general anesthesia is considered to be 45 to 60 [23]. However, we set the adequate sedation goal as a BIS score of 60 to 89. BIS is one of the best tool that reflects the patient’s anesthesia, sedation and alertness. Recent studies have shown that a BIS-guided manual administration compared to a weight-related manual administration of propofol would reduce the incidence of arterial hypotension during induction of general anesthesia [25]. In other words, monitoring of alertness through BIS plays an important role in securing stability of anesthesia or sedation.Most subjects were elderly patients. In addition, all patients had been administered spinal anesthesia. Because deep anesthesia was not required, our BIS score goals were somewhat higher than those of general anesthesia. Hong et al. [14] reported that the use of DMT resulted in longer PACU stays compared to those in the control group comprising of patients who were 65 years and older which used normal saline and were administered spinal anesthesia with low dose of bupivacaine.Therefore, we assumed the adequate sedation level using spinal anesthesia for surgery as light (BIS_80_) to moderate (BIS_70_) sedation and superficial anesthesia (BIS_60_). Using spinal anesthesia, the rate of adequate sedation was not significantly different between the two groups. As mentioned, adequatesedation and anesthesia with spinal anesthesia was based on BIS_80_, BIS_70_, and BIS_60_. These ranges considered sedation and/or superficial anesthesia rather than the depth of anesthesia suitable for surgery. This was related to the nature of DMT used as an adjuvant anesthetic agent rather than the main anesthetic agent. Some subjects did not achieve an appropriate anesthetic level (BIS_50_) during 30 min of measurement. Our results suggested that the long-term administration of tamsulosin does not affect the induction of anesthesia using DMT or the maintenance of anesthesia. It is impossible to compare the time to appropriate anesthesia depth because most hospitals do not use general anesthesia for many TURP or HoLEP procedures. Although our results have measured the effects of selective α1 blockers on the anesthetic use of DMT, it is incorrect to interpret these measurements as differences in speed or depth of adequate anesthesia for surgery.

The most common side effects of DMT are hypotension and bradycardia [26]. When DMT is used with spinal anesthesia, the frequency of such side effects may increase. In this study, no case of hypotension required rescue drugs; however, bradycardia that required rescue drugs occurred in three patients in Group N and one patient in Group T. It is likely that the frequencies of bradycardia and hypotension do not increase when DMT is administered with spinal anesthesia.

Our results showed that HR did not indicate any significant difference in both groups; however, they did show a significant difference in MBP after 15 min. This was an unexpected result. Tamsulosin is a α1A blocker. Furthermore, α1A adrenergic receptors are present in the smooth muscle of the aorta and prostate gland. Therefore, these results suggest that long-term administration may have affected receptors in the smooth muscle of blood vessels even though they did not affect brain receptors. However, this may have been the result of a more complex process. Unfortunately, we cannot conclude any facts from our results. We only know that it is necessary to conduct a new study based on these results.

There were some limitations to our clinical study. The first limit was the age of the subjects. In many clinical studies, the elderly and adults are separated by age (65 years or older and younger than 65 years) for observation of the response to drugs. However, our subjects were BPH patients, and most of them were characterized by onset after age 45 years [10]. Therefore, subjects involved in our study were 45 to 80 years old, and we were forced to compare patients and controls accordingly. The second limitation was that the number of allocated patients in each group was rather small. Patients with BPH at our hospital are prescribed many kinds of selective α1 blockers according to the characteristics of each disease or to the doctor’s preference. Therefore, we will continue this study with patients using tamsulosin and other α1blockers. The final limitation was that there was no follow-up of the recovery time of the patients after sedation or the recovery rate of consciousness. We think it would have been better if the patients’ conditions, recovery times in the PACU, and times to ambulation were measured; however, some of these measurements were impossible. Nevertheless, our study has an important effect to understandthe inter-relationships between DMT and tamsulosin, which are frequently used for patients with BPH, and for understanding DMT, which is currently used as an anesthetic agent, despite some limitations.

## Conclusions

The use of α2 agonists can be considered during surgery for BPH patients using long-term tamsulosin.Patients who have been ontamsulosin for long periods have maintained alertness with the use of α2 agents similar to BPH patients who were not on tamsulosin.

## Additional file

Additional file 1: The raw data of this study. (XLS 28 kb)

## Abbreviations

BIS: Bispectral index
BPH: Benign prostatic hyperplasia
DMT: Dexmedetomidine
FDA: Food and Drug Administration
HoLEP: Holmium laser resection of the prostate
HR: Heart rate
ICU: Intensive care unit
IPSS: International Prostate Symptom Score
MBP: Mean blood pressure
MOAA/S: Modified Observer’s Assessment of Alertness/Sedation
PACU: Postanesthetic care unit
TURP: Transurethral resection of prostate
